# Population cause of death estimation using verbal autopsy methods in large-scale field trials of maternal and child health: lessons learned from a 20-year research collaboration in Central Ghana

**DOI:** 10.1186/s12982-023-00120-7

**Published:** 2023-02-16

**Authors:** Samuel O. Danso, Alexander Manu, Justin Fenty, Seeba Amanga-Etego, Bilal Iqbal Avan, Sam Newton, Seyi Soremekun, Betty Kirkwood

**Affiliations:** 1 Disease Modelling Research Group, Centre for Dementia Prevention & Centre for Clinical Brain Sciences, University of Edinburgh, Edinburgh, UK; 2 Centre for Maternal and Newborn Health, Liverpool School of Hygiene and Tropical Medicine, Liverpool, UK; 3 Faculty of Epidemiology and Population Health, London School of Hygiene and Tropical Medicine, Liverpool, UK; 4 Centre for Computing, Kintampo Health Research Centre, Ministry of Health, Kintampo, Ghana; 5 Faculty of Infectious and Tropical Disease, London School of Hygiene and Tropical Medicine, London, UK; 6 School of Public Health, Kwame Nkrumah University of Science and Technology, Kumasi, Ghana; 7 Faculty of Epidemiology and Population Health, London School of Hygiene and Tropical Medicine, London, UK

**Keywords:** Verbal autopsy, Verbal post-mortem, Maternal neonatal and child health, Child health, Neonatal mortality, Maternal mortality, Child mortality, Vital registration, Death registration, Vital statistics

## Abstract

Low and middle-income countries continue to use Verbal autopsies (VAs) as a World Health Organisation-recommended method to ascertain causes of death in settings where coverage of vital registration systems is not yet comprehensive. Whilst the adoption of VA has resulted in major improvements in estimating cause-specific mortality in many settings, well documented limitations have been identified relating to the standardisation of the processes involved. The WHO has invested significant resources into addressing concerns in some of these areas; there however remains enduring challenges particularly in operationalising VA surveys for deaths amongst women and children, challenges which have measurable impacts on the quality of data collected and on the accuracy of determining the final cause of death. In this paper we describe some of our key experiences and recommendations in conducting VAs from over two decades of evaluating seminal trials of maternal and child health interventions in rural Ghana. We focus on challenges along the entire VA pathway that can impact on the success rates of ascertaining the final cause of death, and lessons we have learned to optimise the procedures. We highlight our experiences of the value of the open history narratives in VAs and the training and skills required to optimise the quality of the information collected. We describe key issues in methods for ascertaining cause of death and argue that both automated and physician-based methods can be valid depending on the setting. We further summarise how increasingly popular information technology methods may be used to facilitate the processes described. Verbal autopsy is a vital means of increasing the coverage of accurate mortality statistics in low- and middle-income settings, however operationalisation remains problematic. The lessons we share here in conducting VAs within a long-term surveillance system in Ghana will be applicable to researchers and policymakers in many similar settings.

## Background

The World Health Organization (WHO) recommends Verbal autopsy (VA) as a method of ascertaining causes of death (CoDs) in low and middle-income country settings (LMICs) [48]. In such settings, coverage of birth registration and medical certification of deaths is often low, and it is consequently difficult to otherwise understand national patterns of public health successes and bottlenecks [40, 45]. Verbal autopsies can provide much needed information on the distribution and burden of disease [20, 45], and can inform the formulation and/or evaluation of health policies and interventions [46].

Verbal autopsies are conducted by trained interviewers with a family member or caregiver of a deceased person. The interviewee will usually have been present with the deceased in the period leading up to the death and will be required to recount details of the health and life circumstances of the deceased in that period. These “autopsies” are then independently reviewed by one or more physicians who assign a cause of death (CoD), or since 2006 automated methods to assign a cause of death provide additional options using standardised data entry forms [33]. In ascertaining CoDs as part of research activities in LMICs, several issues may arise around the processes involved from the collection of the data through to the assignment of the final causes. Key areas that require special attention include the appropriate training of interviewers, cultural adaptation of tools, the effect of diminishing recall of family members of the circumstances surrounding the death over time, the advantages and disadvantages of physician coding and the quality of data, the value of open-ended and closed-ended questions in VA questionnaires, and the use of free-text narratives.

Whilst the WHO provides a set of useful tools for researchers conducting field collections of VA data [56], the purpose of this paper is to share key challenges and perspectives on specific aspects of operationalising VA surveys relevant to maternal and child health research; we focus particularly on lessons we learned optimising the conduct of VAs in the field and the procedures associated with the assignment of cause of death.

VAs were conducted in the Brong Ahafo Region of rural central Ghana as part of nearly two decades of maternal, newborn and child health (MNCH) research in a collaboration between the Ghana Health Service and the London School of Hygiene and Tropical Medicine. A series of cluster-randomised controlled trials (WHO/CHD Immunisation-Linked Vitamin A Supplementation Study Group 1995, [2–4, 26–31] tested the impacts of Vitamin A supplementation and community-based interventions on maternal neonatal and child health, informing international policy for interventions targeting these groups (Soremekun and Kirkwood, to appear). The effectiveness of the MNCH interventions was evaluated primarily as the impacts on maternal, neonatal, and/or infant mortality, resulting in the performance of over 5000 VAs. By the year 2002, more than 200,000 women of reproductive age and their newborns were being monitored under 4-weekly surveillance, making it to our knowledge the largest non-national surveillance system in West Africa. We finally discuss present and future developments in the processes involved in ascertaining CODs within research and programme settings and situate it in the context of current state of advancement in technology and computational methods.

### Tools and questionnaire design

There is extensive literature describing the process of adaptation of a VA form to both the local context and to the particular subgroup in question [49–56]. In our case, our formative research included understanding local or colloquial terms for specific mother and infant–related conditions including references for major complications and risks for mortality.

We focus here on the use of the ‘open history’ in a VA form, as a tool to improve the completeness and chronology of relevant information related to the death. The World Health Organisation (WHO)-VA tool includes space for an optional short open history that can be administered at the end of the interview, and provides basic instructions for potential users. Open histories are free-narrative text descriptions of the circumstances leading to the death, which can be recorded in addition to closed-ended questions on specific topics. Open histories can provide coherent, non-prompted, chronological accounts of the circumstances leading to the death. These texts are potentially rich in information that may otherwise not be elicited in close-ended questions [13]. In the initial phases of the adaptation of WHO VA tools for the ObaapaVita and Newhints studies there was a strong consensus amongst researchers and lead physicians involved in coordinating clinical reviews of the VA forms for assigning causes of deaths (authors AM, BIA, and SN) that open histories were key valuable components of the VA tool with advantages as described above. Part of this consensus was the view that open histories provided a means to gently and slowly lead the respondent into a discussion about the recent death in way that was culturally appropriate and perhaps more acceptable than direct questions about morbidities and symptoms. Open histories however can be time consuming or difficult to follow, and there are conflicting reports of whether the information has negligible [19] or appreciable [35, 22, 32] impact on the quality of the processes of assigning causes of deaths. This debate accounts for newer coding methods which focus on automated algorithms assigning causes of death based on closed-ended questions only [11, 16], which may be less resource intensive than physician review-based approaches. In this section of the paper we discuss our experience of the use of open histories, the contexts in which they may be valuable and their contribution to optimising our VA data collection and review processes.

#### Lesson 1: the use of open histories in VAs can be valuable particularly when cause of death is based on physician review

We trained our interviewers to begin the VA interview by asking the respondent to provide a narrative record of the circumstances leading to the death in question, followed by the administration of close-ended questions and copying of any medical records available for the deceased. The rationale for this approach was to ensure cultural appropriateness, and chronological coherence in the account. Cognisant of the risk that some details might be lost because the respondent might not understand the relevance of or remember to mention each relevant sign and symptom experienced by the deceased if not prompted, we evolved from a single long narrative section to a semi-structured series of narratives that split the time prior to the death into shorter chronological periods (infant VA: pregnancy, delivery, post-partum, chronological events surrounding illness. Maternal VA: Pregnancy (if relevant), delivery (if relevant), post-partum (if relevant), chronological events surrounding illness). The interviewee was prompted at the beginning of each narrative sub-section about specific events and periods around the death.

Cultural sensitivities were important: In the Ghanaian setting, it was customary for visitors to the family of the deceased to sit with the family, accept a drink and listen as the details of the death were recounted by a family member before asking questions or offering further condolences. Thus, after team discussions, we chose to conduct the open histories before beginning close-ended questions to better mimic this natural scenario. Whilst this was a major change from the structuring of the WHO VA tool [55, 56], this also closely simulates the normal experience of patients during clinical consultations, which invariably start with the patient narrating their health problem before the clinician askes a series of follow-on questions. Finally, our experience during training sessions was that direct questioning about signs and symptoms of illness could influence the narrative whereby the respondent might attempt to provide a summary of the symptoms already discussed which could result in loss of coherence or loss of key contextual information not collected in the closed-ended section. Physician-coded VAs from the NEWHINTS study were digitised for a sub-study to develop computational methods for automatic coding of causes of deaths without the need for physician review [15]. The digitisation allowed us to describe key characteristics of 976 VAs from this period. We observed that VA forms originally physician-coded as having an indeterminate cause of death had on average 49 (95% confidence interval: 35.0–63.1) fewer words in their open histories compared to those which were successfully assigned a cause. Whilst there may undoubtedly be other contextual factors which contribute to the variation in narrative length and/or probability of assigning an indeterminate code, which warrant further exploration, this exploratory finding is in line with the views of our coding physicians of the value of the open history section. The successful determination of a cause of death will be only partially indicative of the overall quality of a VA form however; the validity of the final cause is also key. This is more difficult to assess in the absence of a true gold standard against which to compare the accuracy of assigned causes; this remains an ongoing area for debate [10, 25]. A comparison study was performed between causes assigned via VAs in the Ghana MNCH surveillance system with their equivalent death data from local hospital records for a subset of participants (Shannon et al. 2021). The study found good agreement between the two sources for most patient subgroups other than stillbirths. As part of the Amanhi Study, an international WHO-coordinated study of MNCH deaths in 8 countries including the Ghana site, the procedures, use of narratives, and patterns of deaths coded within the Ghana surveillance system were reviewed and optimised including comparison to other sources of mortality data for the country to ensure these remained broadly aligned [3] and have since been published widely [1, 2]. We highlight these outputs to show that the implementation of techniques to increase opportunities to assign a cause of death are to be welcomed, and the inclusion of well structured free text sections in VA forms can be a valuable tool to do this. However, such techniques should be undertaken within the context of overarching protocols that also maintain or monitor the accuracy of causes of mortality themselves. Newer automated methods of assigning causes of deaths, including the WHO-supported InterVA method [11] tend not rely on open histories, instead using algorithms to predict causes of death based on closed ended questions (see section Interpretation of Verbal Autopsies to Assign a Cause of Death). However emerging evidence suggests that algorithms that can capture information from open histories can provide valuable additional information to the coding process [15, 25].

### The verbal autopsy data collectors

#### Lesson 2: VA data collectors may need additional skills that are context-specific.

We agree with the WHO recommendation that VA data collectors be local, acceptable to the community and have at minimum a secondary school education [55, 56] with some caveats. Key attributes for our studies were therefore that our staff had completed high school, had fluent English and a good understanding of the local language and crucially the ability to translate the local language narratives into English-the official national language. This ability to translate was very useful in capturing the open history where the interview was conducted in the local language and transcribed directly into English onto the VA form by the data collector, and may be a desirable trait in similar settings [41].

#### Lesson 3: training in qualitative data collection is essential complement for VA interview skillset

In the first rounds of VA data collection, we observed considerable variability in the quality and length of the VAs and the open history transcripts in particular that had a significant and negative impact on the ability of coding physicians to ascertain a cause of death. Consequently, and with the premium we placed on open histories, later rounds of training of VA data collectors had a major focus on qualitative data collection methodology. This latter component of the training took over two-thirds the total training duration, focussing on developing the communication and listening skills of the VA data collectors; rapport building to secure respondent trust to provide detailed and reliable information; the importance of body language and non-verbal communication; detecting and probing for inconsistencies without appearing judgemental; how to handle issues around privacy and confidentiality; and the need to sympathise and empathise with families and respondents. Common terms used in describing illnesses were discussed and translated from the local language into English by the trainees with support from the trainers. The training also encouraged the data collectors to include verbatim quotes of words and phrases, in the local language, used by respondent to describe medical conditions. This will prevent misrepresentation, loss of meaning or ambiguities in the translation of medical conditions and concepts that may result from attempting to translate these local words into English. For instance, depending on the context, “anidane” is a local term that could represent amenorrhoea, early pregnancy, dysmenorrhoea or irregular menstrual intervals. A blanket translation into one of these may be misleading in the interpretation of VA data by physicians. This qualitative skills component was permanently embedded in the VA data collector training package.

### The respondent and interview

#### Lesson 4: choosing appropriate interviewers may be include approaches to non-family members.

The choice of respondent to interview for a VA is the critical determinant of the quality of the VA. We looked for the most reliable informant and interviewed the person. This informant was usually person who was familiar with and/or socially close to the deceased around the time of the death and was capable of providing chronologically logical, coherent, and reliable information on the circumstances around the death. Although the primary caregiver for the deceased is the obvious choice [55, 56] and was the most popular choice in our studies, our experience taught us that ‘social closeness’ did not always apply to a parent or sibling of the deceased. VA interviewers were therefore trained to make an assessment whether additional information on events surrounding the death might be available from a connection outside the family (e.g. a friend), if there was significantly limited data available from family members. Examples of the type of information might include additional information on events directly leading to the death, or in much rarer cases non-disclosed pregnancies or abortions. Nonetheless, we experienced a larger degree of success when the approach to a friend or alternative family member was brokered by the family in order not to affect research worker-community or family relations. Where adequate rapport is established, families can volunteer a friend to respond to VAs because they know the person will provide the best additional information..

#### Lesson 5: the mourning period, time lapse between death & interview impacts on data quality

Many communities, ethnic and religious groups around the world observe a period of mourning after the death of a member [23]. It is therefore considered culturally insensitive to visit families and conduct an interview within that period despite the value of shorter recall, as this could cause distress or influencing the willingness and ability of respondents to engage in the VA interview process [48]. The WHO’s VA field manual suggests that recall periods ‘longer than 1 year’ should be interpreted cautiously, however detailed information about recall periods beyond this are scarce in the literature. Due to the structure of our 4-weekly surveillance system our staff usually reached families between 6 weeks and 3 months after the death (lag phase) to conduct the verbal autopsy—this can be considered a fairly conservative wait period. We relied on the rapport developed between our field staff and the community. We found that deaths amongst members of nomadic groups or migrant farmers were especially hard to capture, particularly where the death resulted in the family’s migration out of the study site. The WHO VA manual nonetheless recommends VAs are not conducted more than a year after the death due to the risk of inaccuracy (WHO 2012).

### Factors impacting on data quality in the processing of verbal autopsy data

#### Lesson 6: optimising data quality and use I: options available for processing and archiving of closed question data

Data processing for close-and open-ended questions differ. The close–ended component of the VA data employed standard data processing methods using paper-based or tablet software-based data collection. For the paper-based approach used in Ghana, we conducted standard protocols to improve accuracy in transfer of paper-based data into a digital format such as double data entry, verification and range & consistency checks for each question in the close-ended component.

#### Lesson 6: optimising data quality and use II: options are also available for processing and archiving of open history data

Various options are available to digitising and archiving of open histories. One option would be to scan the open history part of the forms and archive these to be made available to physicians for assigning the CoDs. This method of digitising is easy to implement and relatively less resource-intense in terms of human, cost and time. In Ghana, we transcribed the open histories into machine readable transcripts in order to be able to develop and test computational-based text analytics [14].

### Interpretation of Verbal Autopsies to assign causes to death

#### Lesson 7: choice of method for reviewing VA forms and impact of this choice on determination of the final cause of death

We employed the Physician Certified Verbal Autopsy (PCVA) approach to ascertaining the cause of death from the VA questionnaire—process we refer to as “coding” because it was the only option available at the time. Whilst automated methods are available, PCVA remains the most widely used approach [38, 48] and involves employing physicians to manually review the VA questionnaire and assign the probable CoD based on responses provided. Nonetheless questions have been raised as to whether this method is the best use of physicians’ time, produces reproducible results, is cost-effective or time-efficient [20, 27]. Computational or automated approaches are therefore also often recommended, though as far as we are aware, currently no single computational approach has yet been comprehensively demonstrated to be a fully adequate replacement for PCVA [34]. The expertise and skill required to set up and maintain computational coding processes in many low- and middle-income settings is not always available, and in-field testing of computational methods demonstrate that performance is still far from optimal [37]. Physician coded deaths are still the most used standard for training and automation of software-based approaches [25].

**Fig. 1:**
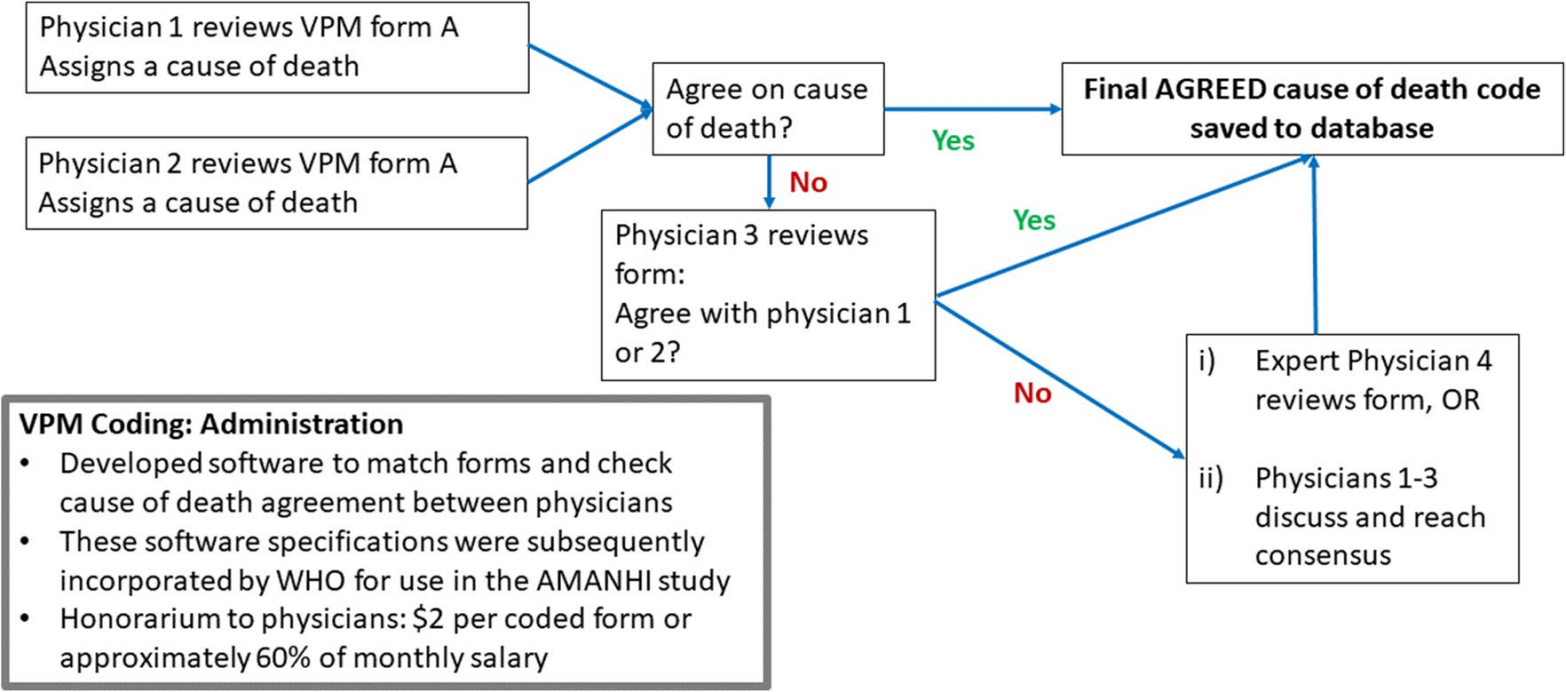
Physician certified verbal autopsy (PCVA) coding process

Figure 1 shows the PCVA process we employed to determine the final cause of death of a woman or child, following several adaptations to the algorithm over the life of the surveillance system. As the figure shows we employed an initial *coding and matching* process where a minimum of two coders did the first round of coding and third coder was only used when there was a disagreement. When all three coders do not agree on a common CoD, the form was elevated to a second stage of the consensus-building process where either a *4th coder* stage also independently coded the forms or a consensus building meeting is held where the coders of the given form discuss and agree or disagree on a common CoD. The Information Box shows the average proportions of VAs where causes of death were agreed by two or more physicians, highlighting the value of the third and fourth coder in improving the overall rate of success in assigning a cause of death in a not insignificant number of cases.

In the 4- coder approach, the fourth coder was a physician or consultant with significantly more experience in maternal and or child health, who reviewed the VA form and codes assigned by the 3 previous coders and then makes a determination on which of the codes should be the final code. Whilst both approaches have significant merits, we evolved from the meeting model to adopt the 4-coder approach because we felt the meeting might force physicians to agree on a cause which they might not have independently agreed on, and it required that all three coders be present at a time and was time and resource-intense. With both approaches, where consensus is not reached, the form was coded as indeterminate.

#### Lesson 8: physician coders: the importance of post-medical training experience

In Ghana, physicians used for the PCVA had a minimum of 1 year post-medical training experience in the care of mothers and babies. To optimise relevance and accuracy, coding manuals were developed and used for the training of physicians by research paediatricians and maternal health experts who also had previous experience in VA coding. The adapted WHO manual used classification principles from the International Classification of Diseases version 10 (ICD-10; 1992) to assign CoDs for women of reproductive age and the Neonatal and Intrauterine death Classification according to Etiology—NICE [52] and the WHO Neonatal Child Health Epidemiological Reference Group—CHERG [9] guidelines for stillbirths and infants. Within this detailed framework, we laid greater emphasis on the principles, and selected sets of causes of deaths that were of public health and programmatic importance within the context of LMIC settings. Where VA data did not allow for assignment of an exact cause, the type of death was classified (e.g. as stillbirth or neonatal death) since from the programmatic point of view, we considered that having information about the type is vitally important and more reliable than being without any information within the context of VA. It is also particularly useful for epidemiological studies [42].

In one of the trials embedded within the LSHTM-Kintampo Health Centre Collaboration surveillance system of the impact of home visits to pregnant women and new mothers on neonatal mortality in central Ghana [30], our first attempt at coding VAs used a mix of newly qualified doctors (up to 3 years post-qualification) from some of the major teaching university hospitals in the country. Upon review of the study results, questions were subsequently raised by the trial steering committee regarding the unusually large proportion of deaths coded as neonatal sepsis, which was higher than the rate of sepsis deaths recorded in the Ghana 2008 District Health Survey DHS [17]. As a result, all neonatal deaths were re-coded, which for logistical reasons took place in the United Kingdom (UK using a mix of experienced UK and Ghanaian doctors with significantly more years of practice post-qualification. As well as standard VA training, an additional peer-sharing workshop was held with the UK-based physicians to outline specific cultural descriptions and terms. The final patterns of causes of neonatal mortality from the second round of coding was considerably more consistent with neonatal outcomes from other national and internationally coordinated studies in the region. Whilst the value of cultural familiarity or knowledge is undoubtably essential, this highlighted the value of general medical experience particularly in the review of clinical signs and symptoms for conditions like neonatal sepsis, which may be poorly understood by caregivers and more ambiguously recalled. The implications extend beyond a need for accuracy in any single study, this experience underlined the importance of minimising cause of death misclassification as a way to accurately record changes in cause-specific mortality fractions over time. We suggest therefore that physician coding of VAs should be optimised where possible by the use of more experienced physicians (minimum 3 years post-qualification with knowledge of/training in the local environment. Additional quality control reviews of a proportion of District Health Survey (DHS with agreed final codes by experienced physicians/specialists is also recommended. Researchers may also want to add more explicit criteria for coding physicians with regards to medical speciality and number of years of experience that seem reasonable within the study context.

#### Lessons for the future: coding: accuracy and future uses of automated method*s*

**Table:** 

**Fig. 2:**
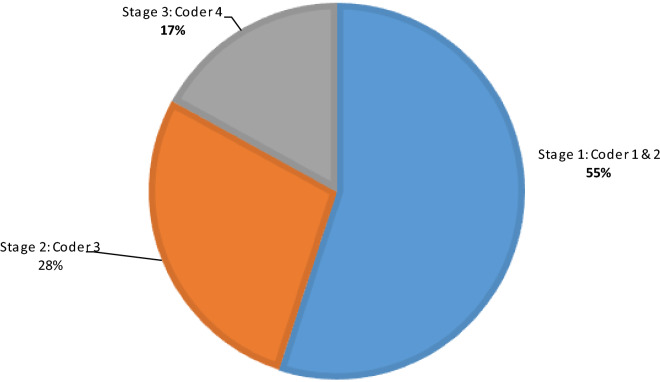
Proportions of 979 NEWHINTS infant verbal autopsies progressing to each stage of the coding process. **Stage 1**: Cause of death (CoD) agreed by two independent physicians, no further review required; **Stage 2**: first two physicians did not agree, a third physician independently reviewed the verbal autopsy form and agreed on the final cause win one of the initial two physicians – no further review required; **Stage 3**: the third physician did not agree a cause with either of the initial physicians therefore either all three physicians meet and discuss until a consensus is reached, or a fourth coder (usually a specialist) reviewed the form (Also described in Fig. 2 graphic). Note this final stage did not always guarantee that a cause of death was finally assigned (a proportion of deaths would remain undetermined)

*Information box: Proportions of coder agreement on final cause of death in 979 infant verbal autopsies in the NEWHINTS Study in Central Ghana 2008–2009*	
Figure 2 shows the final cause of death code (CoD) for 55% of the VA questionnaires were based on the agreement of two physicians). 28% of the questionnaires required a third physician coder to to determine a final CoD, and 17% of the VAs had to be coded by consensus of all three physicians or a 4th coder as described in Fig. 1 above. It is therefore essential to bear this in mind during planning and budgeting for a coding activity

A number of computational methods have been posed in an attempt to address some of the resource and reproducibility issues associated with PCVA as enumerated above. Computational methods such as Tariff 2.0—Smart VA [24]; InterVA [18, 19] and InSilicoVA [36] have been trained and tested on 2016 versions of WHO VA questionnaire, and made publicly available as softwares to support VA analysis [57]. Furthermore, numerous computational methods have been published in the literature which have the potential to revolutionise VA analysis, and this include Quigley et al. [43] based on logistic regression, King and Lu [28] and Murray et al. [39] based on probabilistic modelling. Artificial Intelligence and Machine Learning approaches have also been proposed and this include Danso et al. [14], which is based on Support Vector Machines, and focuses primarily on the open history but also able to combine both coded response and open-history, Jeblee et al. [25] proposed a interpretable Machine Learning model with a focus on usefulness of the open history. Blanco et al. [7] have also taken this further and proposed a Deep Learning approach.

While we do acknowledge that the WHO recommend a number of softwares particularly those mentioned above [55, 56] for automated analysis of VA, we have nonetheless as highlighted previously, for several valid reasons that PCVA may remain an attractive method in many sub-Saharan African countries in the next couple of years [49].

## Conclusion

Birth and death registration is a vital tool for national and international monitoring, and policy-making for population health and socioeconomic wellbeing. Currently however, the status of vital registration is either unknown or below 50% in most countries in sub-Saharan Africa and the Indian subcontinent - the lowest rates of registration globally [UNSD, 2021; UNSD, 2014; BPDHI, 2018]. In this paper we describe the lessons learned in improving the practices and procedures in the operationalisation of VA data collection and cause of death coding. Over the 20-year collaborative project between the LSHTM and the Ghana Health Service’s Kintampo Health Research Centre, our experience indicates that the key processes that can be targeted for optimisation include the choice of VA respondent, the collection and use of open histories, and the physician coding of VA forms. We concluded by reflecting on exciting advances in civil registrations systems, including automated and emerging methods for coding of VAs, whilst acknowledging physician-based cause of death coding methods remain widely used and well-regarded.

## Data Availability

The datasets analysed during the current study are available from the corresponding author on reasonable request.

## Abbreviations

AMANHI: Alliance for maternal and newborn health improvement
CHERG: World Halth Organisation Neonatal Child Health Epidemiological Reference Group
CoD: Cause of death
DHS: District health survey
ICD-10: International classification of diseases version 10
KHRC: Kintampo health research centre, ghana health service
LMIC: Low-and-middle-income county
LSHTM: London school of hygiene and tropical medicine
MNCH: Maternal neonatal and child health
NEWHINTS: Newborn home intervention study
NICE: Neonatal and intrauterine death classification according to etiology
PCVA: Physician certified verbal autopsy
WHO: World Health Organisation
VA: Verbal autopsy
